# Thromboinflammation vs. immunothrombosis: strategies for overcoming anticoagulant resistance in COVID-19 and other hyperinflammatory diseases. Is ROTEM helpful or not?

**DOI:** 10.3389/fimmu.2025.1599639

**Published:** 2025-06-19

**Authors:** Lou M. Almskog, Anna Ågren

**Affiliations:** ^1^ Department of Molecular Medicine and Surgery, Karolinska Institutet, Stockholm, Sweden; ^2^ Department of Perioperative Medicine and Intensive Care, Karolinska University Hospital Huddinge, Stockholm, Sweden; ^3^ Department of Clinical Sciences, Danderyd Hospital, Division of Cardiovascular Medicine, Karolinska Institutet, Stockholm, Sweden; ^4^ Coagulation Unit, Department of Hematology, Karolinska University Hospital, Stockholm, Sweden

**Keywords:** immunothrombosis, thromboinflammation, rotational thromboelastometry, COVID-19, coagulopathy

## Abstract

Thrombosis and inflammation are closely interconnected. Systemic inflammation activates the coagulation system, while components of the coagulation system can, in turn, significantly influence the inflammatory response. This process, where the immune system contributes to thrombus formation, is known as immunothrombosis. Conversely, thromboinflammation describes the effect of thrombus formation on the immune system. Various immune cells, including neutrophils and monocytes, play key roles in these processes, as well as endothelial cells, strategically positioned to rapidly detect and respond to invading pathogens. Platelets are also actively recruited, promoting coagulation and releasing procoagulant factors. When the endothelium becomes dysfunctional and acquires proinflammatory and procoagulant properties, it fosters the formation of microvascular thrombosis. The excessive release of proinflammatory cytokines and chemokines further intensifies this cycle, contributing to cytokine storms, as observed in severe COVID-19 cases. This phenomenon exemplifies immunothrombosis and thromboinflammation. Anticoagulant therapy is standard care for venous thromboembolism prevention in Intensive Care Unit patients, with critically ill COVID-19 patients often receiving higher doses. However, variations in individual responses to heparin were observed in COVID-19 patients, suggesting a degree of resistance to anticoagulant therapy. This resistance may be linked to thromboinflammation, where the intense inflammatory response diminishes the effectiveness of anticoagulation. In this context, combining anticoagulants with immunomodulatory drugs has shown promising potential. This review aims to delve into the concepts of immunothrombosis and thromboinflammation, with a particular focus on the complex interplay between the coagulation and inflammation systems and their mutual reinforcement in the context of COVID-19. We examine why standard anticoagulant therapies often proved insufficient in managing hyperinflammatory diseases and discuss potential alternative treatment strategies. Furthermore, we evaluate the potential role of rotational thrombelastometry (ROTEM) in managing immunothrombotic states.

## 1 Introduction

The interplay between the immune and coagulation systems is complex and in certain circumstances, thrombosis plays a crucial physiological role in immune defense. This process, known as *immunothrombosis*, describes the formation of thrombi within blood vessels, particularly microvessels, as part of the innate immune response. It has been proposed that immunothrombosis, where activated immune cells and platelets together with the coagulation system interact to form blood clots, serve as a platform that facilitates the recognition, suppression, and destruction of pathogens, thereby protecting host integrity. However, when dysregulated, immunothrombosis can lead to severe thrombotic events, or progress into pathological states such as disseminated intravascular coagulation (DIC) (1).

In patients with severe COVID-19, the immune system becomes dysregulated and hyperactivated, with the lungs being particularly affected. SARS-CoV-2 is believed to trigger a state of immunothrombosis, leading to the formation of blood clots, especially in the microvascular system. These microvascular thrombi may contribute to acute respiratory distress syndrome (ARDS) and dysfunction in other organs, suggesting that anticoagulation strategies could be beneficial in moderating the immunothrombosis state. Furthermore, the hyperactivated immune response can provoke an excessive release of cytokines, which is likely a major contributor to the organ damage observed in severe COVID-19 cases (2).

*Immune cells.* The ability of immune cells to effectively eradicate bacteria that invade the body’s tissues is crucial for our survival. For invading organisms to successfully infect the host, they must first overcome the intravascular innate immune system, which comprises humoral factors and various immune cells. Through evolution, bacteria have developed various strategies to overcome these defenses, including avoiding detection, sending signals that confuse immune cells, and altering immune cell function.

Several types of immune cells, including neutrophils, monocytes, natural killer T cells, Kupffer cells, and endothelial cells, are strategically positioned within the vasculature to rapidly detect and respond to invading organisms. Among these, neutrophils are the first to be recruited to inflamed sites during an immune response, where their primary functions are to isolate, engulf, and destroy pathogens, making them key effector cells in the innate immune defense against bacteria (3).

*Endothelial dysfunction*. Although immunothrombosis is intended to protect the host, interactions between immune cells and endothelial cells induce the endothelial cells to acquire proadhesive, proinflammatory, and procoagulant properties, leading to endothelial dysfunction. This dysfunctional state is characterized by glycocalyx degradation and increased vascular permeability, which further accelerates the progression of immunothrombosis (4).

*NETs.* An excessive innate immune response plays a critical role in the development of ARDS in severe COVID-19, where SARS-CoV-2 activates lung epithelial cells and resident macrophages, leading to local cytokine production and neutrophil recruitment. Further, these activated neutrophils release a web of DNA-based cytoplasmic material, known as neutrophil extracellular traps (NETs), which serve as a defensive mechanism against invading pathogens. However, NETs can also accumulate and activate platelets and coagulation factors, serving as a scaffold, leading to thrombus formation. In lung tissue from fatal COVID-19 cases, NETs have been found closely associated with damaged alveoli, and complexes of NETs and platelets, as well as thrombi have been observed in lung microvessels. These findings, which result in blood vessel occlusions, are consistent with immunothrombosis and may lead to ischemic damage and organ failure (5).

*NETosis.* The release of NETs, which occurs during a regulated form of neutrophil cell death known as NETosis, is a crucial effector function that mediates the harmful effects of neutrophils. SARS-CoV-2 induces NETosis and subsequent NET formation in both circulating and tissue-infiltrating neutrophils. In many COVID-19 patients, extensive NETosis and NET generation have been observed, leading to significant inflammation and the formation of characteristic NET-induced thrombi, contributing to microvascular obstruction and organ damage.

NETosis and NETs are increasingly recognized as key contributors to vascular injury and immunothrombosis. During NETosis, NETs and their by-products act as direct amplifiers of inflammation, exposing proinflammatory mediators, proteases, cytotoxic enzymes, and autoantigens. SARS-CoV-2 has been shown to induce NETosis and NET formation, leading to the release of free DNA, elastases, and histones. These by-products can trigger surrounding macrophages and endothelial cells to secrete excessive proinflammatory cytokines and chemokines, thereby enhancing further NET formation and creating a positive feedback loop that drives cytokine storms in COVID-19 (6).

The lungs are particularly susceptible to immunothrombosis, due to the abundant presence of neutrophils and platelets. However, autopsies of COVID-19 patients have revealed microvascular thrombi and necrotic injury in organs distant from the lungs, raising the question of whether pulmonary NET-mediated immunothrombosis can affect remote organs. Possible explanations include the leakage of viral particles or host cytokines from damaged lung tissue into the systemic circulation, despite limited evidence that SARS-CoV-2 becomes blood-borne. Additionally, circulating levels of proinflammatory cytokines may not reach levels typically considered harmful to tissues, yet still contribute to systemic effects (5).

*Hyperinflammation.* In severe COVID-19, innate immune cells release pro-inflammatory cytokines such as IL-1, IL-6, and tumor necrosis factor alpha (TNF-α), which intensify the recruitment of additional immune cells and activate the complement system, leading to a hyperinflammatory state. This excessive inflammation, driven by cytokine release, contributes to intravascular coagulopathy by promoting endothelial dysfunction and the activation of platelets, monocytes, and neutrophils. These processes are deeply interconnected, playing a central role in the pathophysiology of COVID-19 and its associated coagulopathy (7).

COVID-19 exhibits a heterogenous clinical presentation, often associated with thrombosis and microangiopathy. There are several competing theories regarding the relationship between the SARS-CoV-2 virus and the tendency for macro- and microvascular thrombosis: one theory suggests that this might be linked to intravascular coagulation, while another proposes that it could be related to complement-mediated thrombotic microangiopathies.

*Platelets.* While platelets are well known for their roles in maintaining hemostasis and mediating inflammation, they also function as immune cells and play a critical role in the formation of immunothrombosis. Platelets express numerous receptors and store hundreds of secretory products essential for their functional responses, including the secretion of proinflammatory cytokines and chemokines. In the pathophysiology of venous thrombosis, platelets are actively recruited to the vessel wall, where they interact with neutrophils and monocytes, supporting coagulation and secreting procoagulant factors. Beyond this, they also contribute to fibrinolysis and vessel resolution, directly driving the progression of the disease (8).

*Antiphospholipid syndrome*. Antiphospholipid syndrome (APS), like COVID-19, is strongly associated with a high incidence of thrombosis affecting arterial, venous, and microcirculatory vascular beds. APS is diagnosed primarily through the detection of antiphospholipid antibodies (APL), which pathologically target proteins bound to phospholipids. Early in the pandemic, APL were identified in COVID-19 patients who experienced cerebrovascular events (9). However, most studies have not confirmed a clear link between APL and macrovascular thrombotic events in COVID-19, despite shared pathological features between the two conditions. APL play a key role in triggering the activation of blood and immune cells as well as the complement system, driving procoagulant and proinflammatory pathways central to immunothrombosis (10).

This review explores the complex interplay between coagulation and inflammation in severe diseases, known as immunothrombosis. We examine the underlying molecular and cellular mechanisms, emphasizing the intricate links between hemostasis and innate immunity in physiological contexts. Additionally, we examine the utility of rotational thromboelastometry (ROTEM) in assessing various aspects of immunothrombosis. Finally, we draw comparisons between APS and COVID-19, highlighting their contributions to immunothrombotic states.

## 2 Literature review

In this review, we conducted a literature search on PubMed using the terms “*Immunothrombosis*” and “*Thromboinflammation*”. The first 10 references for each term were included in the analysis, with articles published between 2013 and 2024. Studies lacking accessible PDF versions online were excluded. Additionally, pertinent references cited in the reference lists of the 20 original articles were included as well.

### 2.1 Immunothrombosis concept

Inflammation-induced thrombosis, referred to as *immunothrombosis*, offers host defense advantages by limiting the dissemination of pathogens within the bloodstream. However, platelets and the coagulation cascade also modulate inflammatory responses through their interactions with immune cells, the endothelium, and the complement system. These interactions can escalate inflammation, leading to a state of intensified inflammatory activity driven by thrombotic processes, known as *thromboinflammation* (11).

The term immunothrombosis was first introduced in 2013 by Engelmann and Massberg to describe how the innate immune system can initiate thrombotic events (1). It involves the interplay between immune cells, coagulation factors, and immune effector proteins, resulting in the formation of microvascular thrombi. These thrombi act as a physical barrier to trap pathogens and prevent their spread while simultaneously activating immune responses (7).

**Figure 1:**
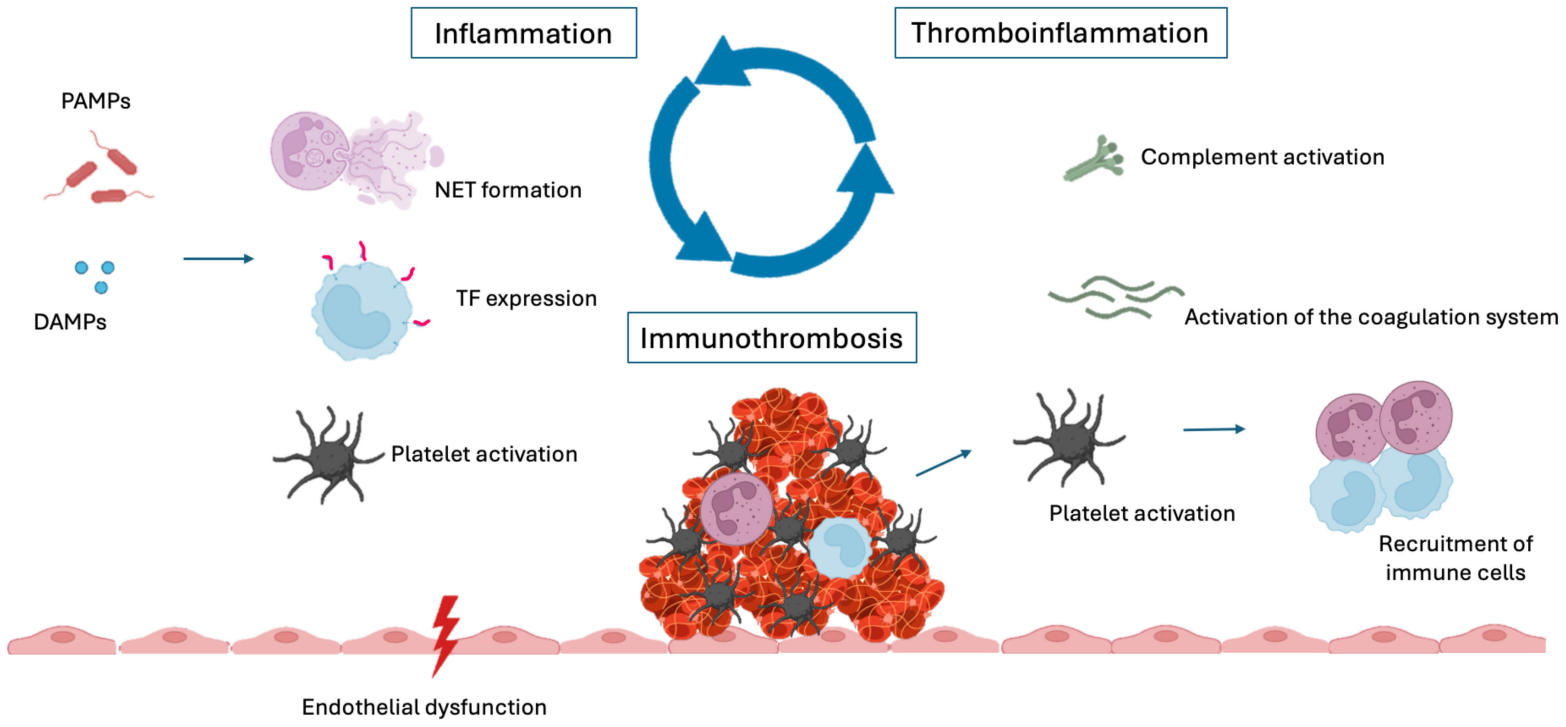
Inflammation, immunothrombosis and thromboinflammation. DAMPs, damage-associated molecular patterns; PAMPs, pathogen-associated molecular patterns; NET, neutrophil extracellular trap; TF, tissue factor.

In contrast, the term thromboinflammation predates immunothrombosis and was initially used to describe the role of platelets in inflammatory processes. Today, the concepts of immunothrombosis and thromboinflammation are recognized as interconnected, with their relationships often understood as a cause-and-effect dynamic: Immunothrombosis refers to the immune system’s role in thrombus formation, while thromboinflammation reflects the reciprocal impact of thrombotic events on immune responses (11) (**Figure 1**).

Cellular signaling plays a central role in sepsis, a condition characterized by profound interactions between inflammation and coagulation. The integrity of endothelial cells is equally crucial, as the loss of their normal antithrombotic and anti-inflammatory functions disrupt homeostasis. This endothelial dysfunction contributes to dysregulated coagulation, complement activation, platelet activation, and immune cell recruitment within the microvasculature (12).

### 2.2 Cellular signaling

Effective control of an inflammatory response relies on precise communication between involved cells, with cytokines, chemokines, and adhesion molecules serving as key mediators of inflammation and immune regulation (13). Cytokines, small glycoproteins released by one cell and recognized by corresponding receptors on target cells, play a central role in inflammation. This diverse family includes interferons, interleukins, growth factors, and chemokines, all of which initiate complex signaling cascades in responsive cells. These cascades drive critical cellular processes such as proliferation, differentiation, and the release of enzymes or additional mediators, which collectively regulate the cellular environment and sustain the effects of cytokines (14).

In sepsis, coordinated communication between leukocytes, platelets, and endothelial cells is essential for the formation of immunothrombosis, where cell adhesion enables intercellular binding and signal transfer. Platelet-leukocyte interactions are initiated by signals from various receptors, including toll-like receptors (TLRs) and protease activated receptors (PARs) (15). Additionally, vascular endothelial cells play an important role in regulating leukocyte trafficking by expressing adhesion molecules. The selectin family of adhesion molecules, including P- and E-selectin on endothelial cells, mediates leukocyte tethering and rolling, while immunoglobulin superfamily adhesion molecules, such as intercellular adhesion molecule 1 (ICAM-1) and vascular cell adhesion molecule-1 (VCAM-1), orchestrate firm leukocyte adhesion (16).

Upon activation, platelets interact with monocytes and neutrophils through key adhesion molecules, particularly P-selectin. When expressed on the platelet surface, P-selectin binds to P-selectin glycoprotein ligand-1 (PSGL-1) on monocytes, facilitating platelet-monocyte adhesion (17). This initial binding is followed by integrin-mediated interactions involving platelet endothelial cell adhesion molecule-1 (PECAM-1), which contribute to scaffolding, signal transduction, and cellular responses (18). Together, these multistep adhesion pathways likely drive the biological and pathological mechanisms underlying thrombosis and inflammation in sepsis (19).

Endothelial cells also release a variety of paracrine mediators, including lipid mediators, nitric oxide, growth factors, and cytokines (20). These signals activate surrounding cells, including monocytes, which upregulate tissue factor (TF) expression under inflammatory conditions. In response, neutrophils release NETs, further promoting coagulation and contributing to the formation of microthrombi, known as immunothrombosis (21). During sepsis, activated leukocytes secrete chemokines and damage-associated molecular patterns (DAMPs) to nearby cells and the adherent endothelium, intensifying inflammatory signaling and exacerbating oxidative stress (22). In COVID-19, endothelial dysfunction, along with the induction of cytokines and growth factors, likely plays a critical role in platelet activation, coagulopathy and thromboembolic complications (23).

### 2.3 Endothelium

The healthy endothelium plays a crucial role in maintaining vascular homeostasis by expressing molecules that are both antithrombotic and anti-inflammatory (12, 24). During sepsis, however, the endothelium becomes activated either directly by pathogen-associated molecular patterns (PAMPs) from bacteria, viruses, and fungi or indirectly through neutrophil extracellular traps (NETs) and proinflammatory cytokines like tumor necrosis factor-alpha (TNF-α), interleukin-6 (IL-6), and interleukin-1 (IL-1) (4). Due to its constant exposure to circulating blood, the endothelium is equipped with mechanisms to counteract thrombosis and inflammation (25). Key examples include heparanoid proteoglycans, prostacyclins, ectonucleotidases like CD39 and CD73, the protein C receptor, and tissue factor pathway inhibitor, which all work to maintain an antithrombotic surface (26). Furthermore, the endothelium functions as a selective barrier, regulating the passage of molecules and cells between the bloodstream and surrounding tissues. When activated, this typically quiescent structure loses its antithrombotic properties and adopts a pro-inflammatory phenotype (10).

Upon activation, vascular endothelial cells interact with leukocytes and platelets, playing a crucial role in immunothrombosis, a systemic response to infections that limits pathogen spread (24). These immune and thrombotic responses are complex, creating procoagulant, proadhesive, and proinflammatory conditions that result in glycocalyx damage, upregulation of adhesion molecules, release of von Willebrand factor (vWF), and vascular tone impairment, all of which facilitate the formation of immunothrombosis (27). When activated, endothelial cells release the contents of their Weibel-Palade bodies, including vWF (28), which interacts with platelet GPIbA, promoting platelet-neutrophil interactions through neutrophil β2 integrins, with evidence suggesting that vWF enhances these interactions. Additionally, CCL5 and CXCL4 (platelet factor 4) have been shown to immobilize on the endothelium, attracting neutrophils and monocytes to sites of thromboinflammation (29).

Vascular endothelial cells also express innate immune receptors, such as toll-like receptors (TLRs) and protease-activated receptors (PARs). Activation of TLRs by agonists like lipopolysaccharides and peptidoglycans upregulates the expression of inflammatory and procoagulant mediators in endothelial cells (30). Additionally, TLR activation modulates microvascular permeability and increases the expression of adhesion molecules (19).

A substantial body of evidence indicates that sepsis-induced endothelial activation leads to increased tissue factor (TF) expression, secretion, and heightened TF activity. Complement components, particularly C5a, have also been shown to stimulate endothelial cells to produce active TF. In addition to TF, other mechanisms contributing to immunothrombosis include the impaired fibrinolytic and anticoagulant functions of the endothelium (4). The process of TF “decryption”, which refers to the crucial post-translational activation step that regulates TF activity, is considered a pivotal factor in driving TF-mediated immunothrombosis (31). Bacterial endotoxins further enhance TF expression while increasing plasminogen activator inhibitor (PAI-1) levels, thereby inhibiting fibrinolysis and creating a procoagulant endothelial surface. Across all cases, endothelial dysfunction is marked by a downregulation of key components of the natural anticoagulant system (12).

Given the important role of the endothelium in regulating hemostasis, fibrinolysis, and vessel wall permeability, endothelial dysfunction in the pulmonary vasculature serves as a trigger for immunothrombosis, leading to the coagulopathy seen in COVID-19 patients. Increasing evidence suggests that COVID-19 is primarily an endothelial disease (32), characterized by elevated levels of PAI-1 and vWF, heightened platelet activation, and a hypercoagulable state, resulting in venous, arterial, and microvascular thrombosis. Although the precise triggers of this endotheliopathy are not yet fully understood, potential factors include direct viral invasion, immune cell infiltration (by neutrophils and macrophages), platelet activation, and hypoxemia, which upregulates TF expression and fosters fibrin-based clot formation, supporting a thromboinflammatory feedback loop. Additional mechanisms may involve complement-mediated damage and surges in pro-inflammatory cytokines, such as IL-1 and IL-6, which lead to direct endothelial injury (2).

SARS-CoV-2 directly infects vascular endothelial cells, causing cellular damage and apoptosis, thereby reducing the antithrombotic function of the normal endothelium (33, 34). In severe cases of COVID-19, endothelial cells in the lung’s blood vessels are further activated by elevated levels of pro-inflammatory cytokines (IL-1, IL-6 and TNF) and ferritin, exacerbating a vicious cycle of immunothrombosis. This hyperinflammatory state induces a hypercoagulable condition, contributing to thrombosis within the pulmonary microvasculature (2).

### 2.4 Coagulation system and tissue factor

The efficient and coordinated activation of intravascular coagulation in response to blood-borne pathogens and circulating products from damaged host cells facilitates the biological process of immunothrombosis. During this process, innate immune cells leverage their procoagulant mechanisms, which include the local release of active tissue factor (TF), the degradation of endogenous anticoagulants, and the formation of a procoagulant matrix composed of extracellular nucleosomes (1). TF is expressed by various cell types, including fibroblasts, pericytes, epithelial cells, monocytes and neutrophils, and also circulates in the blood as microparticles (35, 36). Monocyte-expressed TF, along with TF delivered by monocyte-derived microparticles and possibly neutrophils, is considered the key trigger of coagulation in immunothrombosis (37).

Notably, the activation of intravascular TF is directly linked to leukocyte recognition of pathogens or damaged cells. For instance, the detection of pathogen-associated molecular patterns (PAMPs), such as lipopolysaccharide (LPS), by monocyte receptors (including TLRs and CD14), enhances TF gene transcription and protein expression. Additionally, TF is activated by damage-associated molecular patterns (DAMPs) released from injured host cells. Therefore, intravascular TF activity is upregulated in response to both pathogens and damaged cells through *de novo* synthesis of the TF protein (1).

Upon exposure to the bloodstream following vascular injury, TF forms a complex with activated factor VII (FVIIa), initiating the extrinsic pathway of the coagulation cascade. The release of TF is associated with elevated levels of cytokines and chemokines. The TF-FVIIa complex activates factor X (FXa), which converts prothrombin into thrombin, further amplifying FX activation through the coagulation feedback loop. Thrombin, in turn, initiates the conversion of fibrinogen into fibrin, leading to the formation of a stable clot (7).

### 2.5 Fibrinolytic and anticoagulation systems

The fibrinolytic system, responsible for breaking down blood clots, plays a vital role in regulating inflammation and maintaining the balance between proinflammatory and anti-inflammatory processes. Activated by tissue plasminogen activator (tPA) and urokinase (uPA), this system facilitates the effective resolution of fibrin clots, restoring blood flow following vessel injury. Fibrin serves as the primary substrate for plasmin, promoting the interaction between tPA and plasminogen on its surface, thus enabling its own degradation. Various cell types, including endothelial cells, monocytes, macrophages, and neutrophils, contribute to fibrinolysis by expressing cell surface receptors with fibrinolytic activity, acting as co-factors for plasmin generation, and protecting against circulating fibrinolysis inhibitors. During fibrinolysis, several fibrin degradation products, such as D-dimer, are released and exhibit immunomodulatory and chemotactic functions (11).

Fibrinolysis is primarily controlled by the interplay between tPA and plasminogen activator inhibitor 1 (PAI-1). Upon endothelial injury or exposure to thrombin, tPA is released from the Weibel-Palade bodies of endothelial cells, triggering a surge in fibrinolytic activity. This initial phase of enhanced fibrinolysis, however, is quickly counteracted by increased production of PAI-1 by endothelial cells. The elevated levels of PAI-1 shift the balance away from fibrinolysis, promoting microthrombus formation and contributing to organ dysfunction (38). In sepsis, dysregulated endothelial production of PAI-1 may impair fibrinolysis, promoting thromboinflammation and procoagulant effects (19).

Fibrinolytic proteins are also crucial in regulating the immune response, particularly through their role in clearing proinflammatory fibrin. Plasminogen activators, such as tPA and urokinase, modulate the innate immune response through both fibrinolytic and non-fibrinolytic mechanisms. Plasminogen itself has diverse functions in controlling proinflammatory processes, including the recruitment of monocytes and lymphocytes during inflammation, as well as enhancing macrophage phagocytosis. PAI-1, an acute phase protein, is upregulated in response to bacterial infections, aiding in pathogen clearance and thereby helping to limit inflammation. Endothelial cells produce PAI-1, with its release further increased by inflammatory cytokines (11).

In addition to the fibrinolytic system, the body has an endogenous anticoagulation system that includes tissue factor pathway inhibitor (TFPI), activated protein C, and antithrombin. These natural anticoagulants help prevent widespread systemic coagulation. Like fibrinolytic agents, these proteins also contribute to the immune response. Research has shown that TFPI exhibits antimicrobial properties, while protein C, through the activation of protease-activated receptor 1 (PAR-1), promotes the release of pro-inflammatory cytokines from monocytes, which in turn drives neutrophil chemotaxis (7).

### 2.6 Fibrin and inflammation

Fibrinogen, the key structural component of blood clots, is abundantly deposited in the lungs and brains of patients with COVID-19, where its presence correlates with disease severity (39). Fibrin, the final product of coagulation, not only provides a scaffold for immune cells but also facilitates the recruitment and activation of inflammatory cells (11). Additionally, fibrin contributes to bacterial containment by trapping pathogens within the clot, further enhancing the immune response by promoting chemotaxis and supporting the adhesion of leukocytes, including macrophages, dendritic cells, and neutrophils (7).

It has been shown that fibrin binds to the SARS-CoV-2 spike protein, leading to the formation of proinflammatory blood clots that contribute to systemic thromboinflammation in COVID-19. Additionally, the spike protein disrupts fibrin polymerization, degradation, and its inflammatory properties, suggesting that it delays fibrinolysis. These findings align with observations of dense, fibrinolysis-resistant blood clots in COVID-19 patients, highlighting fibrinogen’s role as a SARS-CoV-2 spike-binding protein that accelerates the formation of abnormal, highly inflammatory clots (39).

### 2.7 Complement

The complement system comprises over 50 proteins that work together through protease activity to enhance immune defenses. These proteins facilitate the recruitment of inflammatory cells, promote opsonization, and aid in the clearance of pathogens, playing a crucial role in both protecting the body from infections and removing damaged cells (40). This intricate network of plasma proteins operates through a cascade of interactions, activated via three primary pathways: the classical, alternative, and lectin pathways. Upon activation, the complement system initiates a series of reactions that culminate in the formation of membrane attack complexes (MACs) on the surface of pathogens or infected cells. These complexes can directly destroy invading microbes, promote phagocytosis by immune cells, and stimulate inflammatory responses, thereby amplifying the immune defense (11).

The complement system can be triggered by various stimuli, ultimately converging at the generation of C5a – a potent chemotactic and pro-inflammatory protein – and the assembly of the MAC, comprising C5b, C6, C7, C8, and C9 (10). There are intricate interactions between the complement system and platelets, which influence both immune responses and coagulation. Upon binding to platelet receptors, complement proteins can initiate a cascade of events that recruit and activate immune cells at sites of injury or infection (11). Platelets express several complement receptors, including cC1qR, gC1qR, C3aR, and C5aR, while complement components like C1q, C3, C4, and C9 can bind to activated platelet surfaces (41). This interaction activates platelets, promoting the surface expression of P-selectin, which facilitates neutrophil adhesion to the endothelium, enhancing immune cell recruitment (42).

The complement system plays a key role in neutrophil recruitment, as proteins like C3a and C5a can upregulate TF activity, further activating neutrophils (7). Additionally, complement activation has been shown to induce rapid TF decryption, increasing its procoagulant function. Complement factor C3 is crucial for platelet activation, and in turn, thrombin can activate both C3 and C5, leading to elevated TF expression on endothelial cells through interactions with C5a and the MAC (C5b-9) (31). Among the complement factors, C3 and C5 are particularly significant in driving thromboinflammation. C3a and C5a promote the production of inflammatory mediators by binding to their respective receptors (38). Moreover, C5a transforms the leukocyte membrane into a procoagulant surface, enhancing neutrophil release of NETs and accelerating coagulation (43, 44).

Complement and coagulation activation are closely linked to COVID-19 severity (31), and abnormal complement activation has been previously associated with thromboinflammatory responses and microangiopathy (45). In severe cases, enhanced activation of the alternative complement pathway correlates with markers of endothelial injury (e.g. angiopoietin-2) and hypercoagulability (e.g., thrombomodulin and von Willebrand factor), highlighting complement activation as a defining feature of COVID-19 (46). Histopathological analysis of skin and lung tissues from patients with severe disease reveals extensive microvascular deposition of terminal complement components, including C5b-9 (membrane attack complex), C4d, and MASP-2, indicative of sustained, systemic complement activation (47). Since complement can both mediate tissue injury and be activated in response to it, the markers of complement activity observed in severe COVID-19 may be both a consequence and a cause of ongoing complement activation (48).

### 2.8 Platelets

Platelets are anucleated blood cells derived from megakaryocytes in the bone marrow. Once released into the bloodstream, they circulate for 7–10 days before being primarily cleared by macrophages in the liver (49, 50). Upon vessel damage, platelets are activated, adhere, change their shape, secrete their granule contents and start to form aggregates (51). Platelets contain three distinct types of storage granules – alpha granules, delta granules, and lysosomes – which house a variety of receptors, soluble proteins, and bioactive molecules. These components play critical roles not only in hemostasis but also in regulating inflammation and immune responses (52).

Platelets are increasingly recognized for their roles beyond hemostasis and thrombosis, particularly in modulating immune responses. For instance, platelet-leukocyte interactions lead to bidirectional immune crosstalk and transactivation, contributing to vascular inflammation (10). Some researchers even classify platelets as immune cells, given their surface expression of pathogen sensors and their ability to enhance immune functions, such as cytokine production and the release of clotting factors in response to stimulation (53).

In addition to their role in initiating and propagating coagulation, innate immune cells, and platelets – key components of clots – play a crucial role in facilitating immunothrombosis through several mechanisms. Platelets promote the recruitment of innate immune cells and enhance the expression of TF, particularly on monocytes. Moreover, activated platelets have evolved antimicrobial strategies that directly support the innate immune response during immunothrombosis and beyond (1). Equipped with a variety of pattern-recognition receptors, including the complete LPS receptor complex, platelets can actively bind circulating bacteria and present these pathogens to neutrophils and other immune cells (54). In response to bacterial products, platelets engage directly with neutrophils, triggering the formation of NETs (55, 56). Although platelets facilitate and accelerate NETosis, NET formation can also occur independently of them (57).

Toll-like receptors (TLRs) are expressed by various immune cells, including platelets, which display TLRs both on their membrane and intracellularly (8). Through these receptors, platelets are able to detect pathogens such as bacteria and viruses. Activation of platelet TLRs triggers not only platelet aggregation but also a pro-inflammatory response. Therefore, TLRs on platelets play a dual role in promoting aggregation and contributing to thromboinflammation (29). TLRs recognize Damage-Associated Molecular Patterns (DAMPs) and Pathogen-Associated Molecular Patterns (PAMPs), leading to cellular activation that generates oxygen and nitrogen radicals and produce cytokines (58). When platelets encounter DAMPs or PAMPs, they become activated and mediate immune responses (8). TLR activation induces immunothrombosis via multiple pathways after PAMP recognition. The surface of activated platelets facilitates thrombin generation, followed by fibrin and clot formation (59).

Platelets have been found to be hyperactivated in patients with COVID-19, potentially exacerbating the thromboinflammatory cascade through interactions with neutrophils. Notably, activated platelets play a critical role in the formation of NETs, which are key components of immunothrombosis. These findings support the hypothesis that endothelial dysfunction and platelet activation are central features of COVID-19 associated coagulopathy, potentially driving the severe damage seen in critical cases (2). This COVID-19-specific platelet hyperactivation, characterized by elevated P-selectin expression and increased circulating platelet–neutrophil aggregates, likely contributes to the heightened immunothrombotic response observed in severe disease (60).

Upon activation, platelets release alpha granule-stored molecules, which contribute to the recruitment of additional monocytes and neutrophils. Monocytes then interact with platelets, forming platelet-monocyte complexes that induce TF expression in monocytes. Meanwhile, hyperactivated neutrophils form platelet-neutrophil aggregates, promoting further NET formation and fueling the cycle of prothrombotic activity (7). This sequence of events, combined with platelet aggregation and fibrin deposition, leads to thromboinflammation and vessel occlusion, even in the absence of direct vascular injury (61).

### 2.9 Leukocytes: neutrophils, neutrophil extracellular traps and monocytes

Leukocytes are critical cellular components in the defense against infections, with neutrophils and monocytes playing key roles in thromboinflammation. While neutrophils and monocytes share many common receptors, their functions differ significantly. Monocytes act as the conductors of inflammation, coordinating immune responses, whereas neutrophils are frontline defenders, directly attacking and immobilizing pathogens.

In response to various stimuli, monocytes become prothrombotic, releasing proinflammatory cytokines and triggering innate immune responses. Activated neutrophils amplify inflammation by releasing proteases and oxygen radicals, ultimately undergoing different forms of cell death, such as necrosis, necroptosis, and pyroptosis. Cellular components released from dying cells, particularly DAMPs like DNA and histones, are highly proinflammatory and procoagulant (62).

Monocytes and neutrophils play critical roles in thrombosis, with many thrombosis-related host molecules being produced or activated by these cells. Activated monocytes, along with the microparticles they release, express intravascular TF, promoting coagulation during thrombus formation. Intravascular TF may also be expressed by other immune cells, such as neutrophils, eosinophils, and platelets, further contributing to clot development. Both monocytes and neutrophils are rapidly recruited to the vessel wall or become incorporated into growing clots (1).

Both neutrophils and monocytes are capable of releasing extracellular traps – web-like structures composed of DNA and intracellular components. While neutrophil extracellular traps (NETs) have gathered the most attention, monocyte extracellular traps (METs) also play a significant role in the body’s defense against infection and are central mediators of thromboinflammation (38). NETs consist of a meshwork of DNA, histones, and antimicrobial proteins that are expelled from neutrophils upon activation (1, 8). In COVID-19, circulating neutrophils release increased levels of NETs in the blood, trachea, and lungs (63). These NETs contain nucleosomal components, including histones, DNA, extracellular viral micro-RNAs, and TF, all of which contribute to immunothrombosis (64).

#### 2.9.1 Neutrophils

Activated neutrophils exert their antimicrobial activity primarily through three mechanisms: phagocytosis, degranulation, and the release of neutrophil extracellular traps (NETs) (4). As the most abundant leukocytes in peripheral blood, neutrophils are rapidly recruited to sites of inflammation, where they play a crucial role in eliminating pathogens. The discovery of NETs has significantly highlighted neutrophils’ involvement in immunothrombosis (49).

Neutrophils possess an impressive range of toxic molecules to kill bacteria. Upon capturing and phagocytosing bacteria, they internalize them into phagolysosomes, where microbial destruction occurs. However, the release of NETs enhances bacterial capture by trapping pathogens more efficiently (3). During NETosis, neutrophils expel their DNA, along with histones and granule-derived enzymes like myeloperoxidase and elastase, to form NETs. This process often coincides with neutrophil death, during which bacteria and toxins are trapped and immobilized in the bloodstream. Thus, while NETs defend against extracellular pathogens by trapping and neutralizing them, macrophage pyroptosis provides defense against intracellular pathogens by killing the host cell, thereby eliminating the pathogen within (31).

Neutrophil maturation significantly impacts NETosis and NET formation, with mature neutrophils generally exhibiting a greater capacity to produce NETs in response to external stimuli. Additionally, a specific subset of neutrophils known as low-density granulocytes (LDGs) is significantly elevated in individuals with severe COVID-19. These LDGs are more prone to spontaneous NETosis and NET production, contributing to immunothrombosis and organ damage in COVID-19 (6).

#### 2.9.2 Neutrophil extracellular traps

For a long time, phagocytosis was considered the primary mechanism by which neutrophils neutralized invading pathogens. However, in 2004, a new process was identified, in which neutrophils release webs of chromatin into the extracellular space, known as neutrophil extracellular traps (NETs) (57). These NETs contain various proinflammatory and procoagulant components, including nuclear DNA, histones, and granular enzymes such as myeloperoxidase and neutrophil elastase. The negatively charged extracellular DNA triggers the intrinsic coagulation pathway by activating factor XII, while histones promote platelet aggregation and activation. NETs also serve as a scaffold for platelet binding (65, 66).

NETosis refers to the programmed cell death of neutrophils, characterized by the formation of NETs (67). This process is triggered in response to both infectious agents, such as bacteria, fungi, protozoa, and viruses, as well as sterile stimuli, including activated platelets, endothelial cells, complement proteins, cytokines, autoantibodies, and immune complexes (68). Although NETs likely evolved to trap and eliminate pathogens, they are now well-recognized for their prothrombotic properties (1). NETs actively promote platelet activation and clot formation and have been detected in both deep vein thrombi and arterial clots (10).

When NETs are released in response to a stimulus, the neutrophil’s membranes dissolve, and its nuclear content decondenses into the cytoplasm. This is followed by the rupture of the plasma membrane, releasing decondensed chromatin along with granular proteins into the extracellular space. In this state, the neutrophil can trap microorganisms and release its intracellular components – such as genetic material, histones, and antimicrobial peptides – onto the surface of the pathogen, leading to its destruction (7).

The NET-associated protein cargos are linked to an increased expression of proinflammatory mediators, including IL-6, IL-8, and CXCR2. NETs enriched with TF have been observed in patients with ARDS, where they are thought to drive immunothrombosis and exacerbate ARDS-related thromboinflammation (31). Additionally, NETs are believed to degrade natural anticoagulants like thrombomodulin and TFPI, further promoting thrombus formation (55). NETs are also rich in prothrombotic factors, and studies have demonstrated that neutrophils in COVID-19 produce TF-carrying NETs, underscoring the role of NETs in the prothrombotic and proinflammatory mechanisms contributing to COVID-19 pathology (6).

NETs have been found to promote procoagulant and prothrombotic activity through several mechanisms. They can act as both a scaffold and inducer for platelet adhesion, activation and aggregation, driven by NET components such as histones H3 and H4. Additionally, NETs stimulate thrombin generation by activating the intrinsic coagulation pathway, likely due to the negatively charged surface of extracellular DNA, which can bind and promote factor XII activation (4). Studies further suggest that NETs contribute to endothelial dysfunction, as evidenced by increased endothelial expression of molecules such as ICAM-1, VCAM-1, E-selectin, TF, and vWF. This dysfunction is also associated with enhanced platelet adhesion to endothelial cells, facilitated by the upregulation of vWF expression on the endothelium (69).

NETs appear to be a crucial feature of immunothrombosis, acting as catalytic surfaces that both promote and compartmentalize the coagulation system (1). They have been recognized as key mediators of tissue damage in inflammatory diseases, particularly through their role in immunothrombosis. In the context of COVID-19, SARS-CoV-2 has been shown to directly stimulate NET release from healthy neutrophils, a process that is dependent on angiotensin-converting enzyme 2 (ACE2) and active viral replication (63).

NETs provide a structural platform that facilitates thrombin generation through the contact activation pathway, promoting microthrombosis. Additionally, NET-induced endothelial damage is closely linked to vascular dysfunction (31). In COVID-19, NETs are thought to be important contributors to dysregulated immunothrombosis, exacerbating the immune response and fueling the hyperinflammation that can lead to severe outcomes and increased mortality. Postmortem analyses of lungs from COVID-19 patients have revealed widespread microvascular thrombi containing neutrophils releasing NETs interwoven with platelets. Elevated NET levels have also been detected in tracheal aspirates from intubated patients, supporting the hypothesis that NET-driven immunothrombosis underlies many of the clinical manifestations of severe COVID-19. Moreover, neutrophils from COVID-19 patients appear particularly primed for excessive NET release, with markers of NET formation correlating closely with disease severity and patient outcomes (63, 70).

It has been shown that SARS-CoV-2 infection can directly induce NETosis and trigger NET release in healthy neutrophils (63). In COVID-19 patients, inflammatory microvascular thrombi containing NET components and platelets have been detected in the lungs, kidneys, and heart (71). NETs contribute to microvascular thrombosis, tissue damage, and organ failure by entering the circulation, binding to vessel walls, capturing platelets and microvesicles, and ultimately obstructing blood flow. Endothelial *in situ* microvascular thrombosis, which may be induced by NETs, is an important cause of thrombosis in COVID-19 (6). Moreover, a deficiency in NET clearance has been identified in COVID-19, leading to pulmonary thromboinflammation (72).

#### 2.9.3 Monocytes

Monocytes, along with macrophages and dendritic cells, are integral components of the mononuclear phagocyte system (MPS) (73). In humans, they account for about 10% of the total leukocyte population in circulation (74). These cells play a crucial role in both tissue homeostasis and immune defense, forming a highly adaptable and diverse cell population. Monocytes are composed of several subsets that differ in phenotype, size, morphology, and gene transcription profiles (75). Each subset has distinct functions: non-classical monocytes are believed to patrol blood vessel walls and support endothelial cells, whereas classical monocytes are capable of crossing the endothelium to enter tissues, where they respond to specific signals and participate in immune responses (29). Monocytes can also differentiate into macrophages, which are capable of phagocytosing pathogens and damaged endogenous particles, while also producing proinflammatory cytokines to amplify the inflammatory response (76).

Monocytes have protease-activated receptors (PARs), a type of G-protein-coupled receptor crucial for mediating inflammation and coagulation in sepsis. They primarily express PAR-1 and PAR-3, which are activated through proteolytic cleavage by their tethered ligands after binding to thrombin (19). Both monocytes and macrophages play important roles in sepsis-induced immunothrombosis, with TF expression by monocytes identified as a primary mechanism driving the coagulation cascade activation in sepsis (4). Monocytes are the main source of inducible intravascular TF, presenting it on their cell surface and releasing it in the form of microparticles. The upregulation of TF expression is central in the development of immunothrombosis (77). Research indicates that pathogens trigger the expression and release of monocyte-derived TF into the bloodstream, which subsequently activates the extrinsic coagulation pathway (4). *In vitro*, monocyte TF expression is upregulated in response to the recognition of DAMPs or PAMPs. Additionally, DAMPs have been shown to enhance TF activity (49).

### 2.10 Therapies

“If we consider COVID-19 as a vascular disease primarily involving the endothelium, an ideal therapeutic approach would be both antithrombotic and anti-inflammatory *- Bonaventura et al. (2)*”.

Various therapies targeting immunothrombosis have been proposed, including treatments that modulate cell-specific immune responses, inhibit platelet-endothelial interactions, or block platelet activation. These range from well-established therapies to more experimental approaches, some of which remain theoretical. Developing treatments that target endothelial innate immune responses and immunothrombosis holds significant potential for reducing morbidity and mortality in sepsis patients (4). However, no drugs specifically targeting immunothrombosis have been approved for clinical use in sepsis, primarily due to inconclusive efficacy results and an increased risk of side effects (4). Ongoing research continues to explore the effects of new molecules aimed at intracellular inflammatory pathways, NET formation, and complement components.

#### 2.10.1 Immunomodulatory drugs

Corticosteroids have previously been linked to an increased risk of venous thromboembolism (VTE) in patients treated for inflammatory diseases. However, it remains unclear whether this elevated thrombotic risk is primarily due to the underlying inflammation or if corticosteroids themselves have prothrombotic effects (78). Corticosteroids were also the first drugs to demonstrate a survival benefit in COVID-19 treatment, with dexamethasone reducing 28-day mortality in patients requiring respiratory support, whether through mechanical ventilation or supplemental oxygen (22.9% vs 25.7%; RR 0.83, 95% CI 0.75-0.93). The study did not specifically address the impact of corticosteroids on thrombosis risk. However, among patients who died, fewer receiving dexamethasone had stroke listed as the cause of death, though the difference was not statistically significant (0.2% vs 0.1%, RR 1.53, 95% CI 0.41-5.71) (79).

Immunomodulatory therapies, including interleukin inhibitors targeting IL-1 and IL-6, granulocyte-macrophage colony-stimulating factor (GM-CSF), and Janus Kinase (JAK) inhibitors, are designed to reduce the cytokine dysregulation seen in severe COVID-19, though their impact on the disease’s thrombotic complications remain poorly understood (2). Cytokine-blocking drugs have yielded mixed results in COVID-19 patients, with outcomes varying across studies. In patients not requiring mechanical ventilation, the IL-6 receptor antibody tocilizumab reduced the risk of progression to mechanical ventilation or death, though it did not improve overall survival (80). Another study found that IL-1 and IL-6 blockers did not shorten time to clinical improvement in COVID-19 patients who had a low baseline mortality risk (81).

However, in the RECOVERY trial, tocilizumab significantly increased the likelihood of hospital discharge within 28 days (57% vs 50%; P <.0001) and reduced the composite endpoint of mechanical ventilation or death (35% vs 42%; P <.0001) in hospitalized COVID-19 patients (82). Notably, 82% of patients in this trial also received corticosteroids, which could explain why tocilizumab showed less consistent benefits in other studies (83, 84). In summary, tocilizumab appears to offer benefits when combined with corticosteroids in COVID-19 patients with hypoxemia and systemic inflammation, who do not yet require mechanical ventilation.

Fibrin-targeting immunotherapy, aimed at neutralizing fibrin toxicity, has been proposed as a therapeutic strategy for neuroprotection and selective suppression of pathogenic inflammation in COVID-19. The monoclonal antibody 5B8, which targets the inflammatory domain of fibrin, has demonstrated the ability to reduce inflammation and oxidative stress in mouse models, suggesting that neutralizing fibrin could offer protective effects in both pulmonary and neuronal tissue (39).

#### 2.10.2 Anticoagulants and antiplatelet drugs

The use of low molecular weight heparin (LMWH) has played a central role in the treatment of COVID-19 (8). However, beyond its well-known anticoagulant effects, heparins also exhibit significant anti-inflammatory properties, which are well-documented (85). By binding to inflammatory mediators and enzymes, heparin suppress the activation of inflammatory cells, thereby modulating the inflammatory cascade and limiting tissue damage (86). Additionally, heparins possess antiviral properties, with clinical evidence suggesting that both UFH and LMWH may reduce mortality in COVID-19 patients experiencing hypercoagulation (87). Heparin and heparan sulphate play a unique role in viral inhibition by directly interacting with the SARS-CoV-2 spike protein. Specifically, their sulphate groups bind to the receptor-binding domain of the spike protein, competing with human cell receptors and reducing the virus’s ability to enter host cells.

Despite these advantages, individual responses to heparin, whether unfractionated heparin (UFH) or LMWH, vary significantly in COVID-19 patients. In some cases, LMWH may provide insufficient therapeutic effects, while in others, its effects may even be counteracted. This variability in heparin sensitivity is likely influenced by factors such as histone levels and heparanase activity, which can influence the effectiveness of heparin treatments (8).

Thrombin plays a central role in coordinating thrombotic and inflammatory responses and has long been regarded as an appealing therapeutic target to reduce thromboinflammatory complications (12). Although anticoagulant therapy is widely accepted in the management of COVID-19, the benefits of anticoagulant and antiplatelet drugs in targeting immunothrombosis in sepsis remain inconsistent, often associated with a significant risk of bleeding complications (4).

According to international guidelines for sepsis and septic shock, anticoagulant or antiplatelet therapy is not recommended for the treatment of sepsis or DIC, except for the use of LMWH or UFH for the prevention of VTE (88). Although some studies have shown a reduction in 28-day mortality with heparin treatment in sepsis, the increased risk of bleeding events remains a significant limitation of its use (89, 90). In non-critically ill COVID-19 patients, anticoagulation is standard therapy for VTE prophylaxis, with higher doses for selected groups, as LMWH/UFH has been shown to reduce VTE incidence and increase organ support-free days (91, 92).

In the early 2000s, researchers investigated the therapeutic potential of physiological anticoagulants, including tissue factor pathway inhibitor (TFPI), activated protein C, and antithrombin, along with their recombinant forms. Due to the shared anticoagulant and anti-inflammatory properties of these key endogenous pathways, many studies have attempted to leverage them for therapeutic benefits in sepsis (31). In Japan, replacement therapy with anticoagulant factors antithrombin and thrombomodulin is even recommended for treating sepsis-related DIC (93). However, despite initial promise, these anticoagulants have not shown efficacy in large-scale RCTs (38).

For many years, platelets have been the primary clinical target in managing arterial thrombotic diseases (94). In contrast, platelets are thought to play a less significant role in venous thrombosis, where platelet-rich clots are not as prevalent (95). As a result, antiplatelet therapy is not typically used to treat recurrent VTE, as its efficacy in preventing secondary VTE remains debated. Instead, anticoagulants, which target the coagulation cascade and inhibit secondary hemostasis, are more effective in managing this condition. Of the platelet inhibitors, only aspirin has been rigorously tested for VTE prevention in clinical studies.

Although aspirin’s mechanism of action remains partially unclear, it has been used for thousands of years as an effective pain reliever and anti-inflammatory drug, primarily due to its ability to block prostaglandin production, which transmits pain signals (8). Despite its well-known anti-inflammatory and antithrombotic effects, a large RCT involving 16 000 sepsis patients found that daily low-dose aspirin had no impact on mortality (96). Similarly, the RECOVERY trial showed no added benefit from combining aspirin with standard anticoagulant therapy in hospitalized COVID-19 patients (97), and another RCT demonstrated no mortality benefit from the P2Y12 inhibitor ticagrelor, when combined with heparin, in non-critically ill COVID-19 patients (98).

#### 2.10.3 Anti-NETs

Anti-NET therapy, which targets the prothrombotic web-like structures formed in the extracellular space, is emerging as a promising strategy for antithrombotic treatment. Given the direct role of NETs in the immunothrombotic processes of COVID-19, blocking their formation may help to improve patient prognosis (2, 8). Studies have shown that both preventing NET formation and dissolving existing NETs can reduce thrombosis in animal models (10). Additionally, NET-driven bacterial capture can be disrupted by anticoagulants or by interfering with the procoagulant components of NETs (1). Encouraging preclinical studies have demonstrated the potential of NET inhibition in mitigating sepsis-induced thrombosis, with DNase administration promoting NET degradation, reducing intravascular thrombin activity, and decreasing microthrombus formation in liver sinusoids (4).

The degradation of NETs appears to be a safe treatment option, as evidenced by drugs like DNase I, which can digest NETs *in vitro* and prevent thrombosis in experimental mouse models. Moreover, the use of heparin or colchicine has shown promise in disrupting NET formation by inhibiting histone-induced coagulation or actin cytoskeleton rearrangement in NET-forming neutrophils. In APS, targeting the interaction between neutrophils and endothelial cells offers another potential way to prevent NETosis and thrombosis (11, 49).

#### 2.10.4 Anti-complement therapy

Growing evidence highlights the role of complement activation in the pathogenesis and severity of COVID-19, particularly its contribution to complement-mediated thrombotic microangiopathy. In this context, complement inhibitors– therapies typically used to treat thrombotic microangiopathy– have been proposed. Blocking C3 can prevent the production of both C3a and C5a, as well as reduce intrapulmonary C3a levels and IL-6 release from alveolar macrophages (2). However, clinical studies on the use of complement inhibitors in COVID-19 have produced mixed results, and the potential benefits of complement inhibition in septic patients remain unproven (38).

#### 2.10.5 Inhibition of tissue factor

New approaches to regulate TF decryption offer another promising target for anti-immunothrombotic therapies. One approach involves inhibiting the TF: FVIIa complex, through inhibitory antibodies directed at this complex, to reduce immune-mediated TF expression and activity. Another potential target is the key cysteine residues involved in TF decryption, where covalent inhibitors have been suggested (31). Additionally, other molecules that inhibit pathways involved in TF expression within immune or endothelial cells, or in its procoagulant activity, have been proposed. For example, inhibitory monoclonal antibodies against CD14 have been shown to decrease TF expression in leukocytes, reducing extrinsic coagulation pathway activation, while also enhancing fibrinolysis by lowering plasma levels of PAI-1 (4).

### 2.11 Coagulation laboratory tests in thromboinflammation/immunothrombosis

Patients with severe COVID-19 who experience thromboembolic events often present with elevated inflammation markers, including CRP, IL-6, fibrinogen, and ferritin, suggesting a strong link to immunothrombosis (99). Immunothrombosis is widely regarded as a central pathophysiological mechanism in the development of sepsis-associated disseminated intravascular coagulation (DIC). DIC is characterized by endothelial dysfunction and an imbalance in procoagulant, anticoagulant, and fibrinolytic systems.

In severe sepsis, elevated plasma levels of thrombin and D-dimer, both markers of coagulation activation, correlate with increased disease severity. Accordingly, decreased levels of natural anticoagulants, such as protein C, protein S, and antithrombin, are commonly observed and are strongly associated with poorer clinical outcomes. Research also indicates that ICU patients with sepsis who exhibit enhanced NET formation are more likely to develop thrombocytopenia, prolonged PT and aPTT, elevated d-dimer levels, and decreased fibrinogen, all of which are predictive of DIC development (4).

In the early stages of the COVID-19 pandemic, certain changes in standard coagulation tests were observed, most notably elevated D-dimer levels and hyperfibrinogenemia (100). D-dimer, an indirect marker of fibrinolysis and fibrin turnover, reflects the systemic breakdown of vascular thrombi through the fibrinolytic process. Studies showed that D-dimer levels rose significantly in the early stages of COVID-19, with a 3- to 4-fold increase linked to poor prognosis (101, 102). Fibrinogen, which is converted to fibrin by thrombin, plays a crucial role as the primary structural component of blood clots. Severe COVID-19 has been associated with significantly elevated fibrinogen levels, leading to excessive fibrin deposition and impaired fibrin degradation (103).

Thrombocytopenia and PT prolongation were uncommon in COVID-19 associated coagulopathy (104). A low platelet count, when observed, was however linked to higher mortality rates (105). Another study revealed a significant increase in vWF activity and decreased ADAMTS-13 levels in COVID-19 patients, suggesting that impaired regulation of vWF and the reduced capacity of its cleavage by ADAMTS-13 could be indicative of an increased thrombosis risk (38).

Viscoelastic testing, mostly employed in trauma and high risk of bleeding surgery to guide resuscitation, has also been used in hypercoagulative states (106, 107). This capability to detect and quantify hypercoagulative states represents a significant advantage of ROTEM over conventional tests (108, 109).

Four key ROTEM variables are commonly analyzed. EXTEM, an extrinsically activated assay using phospholipids and tissue factor, assesses the extrinsic coagulation pathway. INTEM, an intrinsically activated assay with phospholipids and ellagic acid, evaluates the intrinsic pathway. FIBTEM is a fibrin-based assay that isolates the fibrinogen function by incorporating tissue factor and the platelet inhibitor cytochalasin D, which excludes the platelet contribution to clot formation. HEPTEM, is similar to INTEM, but includes heparinase to neutralize the effects of heparin.

Within each ROTEM-variable, five parameters are measured. Clotting time (CT) represents the time to the onset of clot formation, indicating coagulation activation. Clot formation time (CFT) reflects the time required for the clot to reach a 20 mm amplitude, providing information about clot propagation. Maximum clot firmness (MCF) measures the peak amplitude, which assesses clot stability. Clinically, a high MCF is indicative of a hypercoagulable state. Lysis index (LI-30 and LI-60) represents the percentage of clot breakdown occurring 30 and 60 minutes after CT, respectively, indicating the extent of fibrinolysis over time (110–112).

In severe COVID-19, an example of an immunothrombotic state, ROTEM analysis revealed prolonged CT, shortened CFT, increased MCF, and elevated LI60, indicating delayed coagulation initiation, faster clot propagation, increased clot stability, and reduced fibrinolysis. In other words, while clots took longer to form, once initiated, they developed more rapidly, became stronger, and were more resistant to breakdown compared to those in healthy individuals.

Prolonged CT, unlike other ROTEM parameters associated with immunothrombosis, is not indicative of hypercoagulation and presents a somewhat paradoxical finding. The prolongation of INTEM-CT is likely attributed to the effects of heparin, whereas EXTEM-CT prolongation is not. Prolonged EXTEM-CT, similar to prolonged prothrombin time (PT), has been observed in some studies of COVID-19 patients and may indicate deficiencies in clotting factors II, VII, and X, which have been observed in COVID-19 patients (113, 114).

The shortened CFT and increased MCF, indicating a rapid increase in clot strength, are consistent with elevated fibrinogen levels and enhanced platelet activation observed in severe COVID-19 cases (115). These changes are likely driven by the massive inflammatory response that characterizes the disease and may signal the presence of immunothrombosis.

### 2.12 APS

Antiphospholipid syndrome (APS) is a systemic autoimmune disease characterized by the persistent presence of antiphospholipid antibodies (APL) in individuals who experience thrombotic events or specific pregnancy-related complications (49). The APLs included in the APS classification criteria are anti-cardiolipin (ACL), anti-β2 glycoprotein 1 (β2GP1) antibodies, and lupus anticoagulant (LA) (116). APL are more commonly identified in individuals with other autoimmune disorders, particularly systemic lupus erythematosus (SLE) (117). The persistent presence of APL induces a prothrombotic state, predisposing patients to thrombosis. According to the “two-hit” model of APS-related thrombosis, a primary “first hit” disrupts the endothelium and creates a predisposition to clot formation, while a “second hit”, triggered by factors such as pregnancy, infections, or traditional cardiovascular risk factors, drives clinical thrombotic events (118). The cornerstone of APS management is anticoagulation therapy, with vitamin K antagonists being the preferred agents supported by the strongest clinical evidence. However, recurrent thrombotic events remain a significant challenge even in patients receiving adequate anticoagulation therapy (119). In Catastrophic Antiphospholipid Syndrome (CAPS), a condition marked by widespread microthrombosis driven by an increased inflammatory response, management typically involves a comprehensive triple therapy approach. This includes anticoagulants, plasmapheresis, and high-dose corticosteroids, with the addition of intravenous immunoglobulins in selected cases (120).

In APS, the systemic prothrombotic and proinflammatory state arises from the impact of APL on various components of the innate immune system, including monocytes, neutrophils, platelets, and endothelial cells (121). This persistent state, which is not fully addressed by anticoagulant therapy alone, underscores the need for therapeutic strategies targeting the innate immune system, as such interventions may provide additional clinical benefits (94).

Monocyte cytokine production and TF expression play a central role in the pathophysiology of APS. APL activate monocytes, driving them into a proinflammatory and procoagulant phenotype characterized by the production of cytokines such as TNF-α and IL-6, along with TF. Notably, monocytes from thrombotic APS patients exhibit higher TF expression and activity compared with monocytes from APS patients without thrombosis. Neutrophils also contribute to the thrombotic process by releasing NETs in response to APL, which further promotes clot formation. APL-induced NET release drives thrombin generation as well as platelet activation and aggregation, reinforcing the prothrombotic cascade. This process is further exacerbated by increased interactions between neutrophils and endothelial cells, mediated through P-selectin glycoprotein ligand-1. Additionally, platelet-leukocyte interactions are enhanced, likely driven by elevated levels of soluble P-selectin and soluble CD40-ligand, further amplifying the prothrombotic state (49).

The enhanced release of NETs in APS is supported by observations that neutrophils from APS patients spontaneously release NETs, with APL targeting β2GP1 significantly amplifying this process (122). NET accumulation in APS is further exacerbated by a reduced capacity to degrade these structures, linked to the presence of anti-NET antibodies. These antibodies are associated with recurrent VTE, emphasizing their role in the disease’s thrombotic burden (123, 124). Taken together, these observations suggest that the dual mechanisms of exaggerated NET formation and impaired NET degradation synergistically intensify the prothrombotic effects of NETs in APS (10).

In COVID-19, elevated APL titers have been associated with increased neutrophil and platelet activity, correlating with more severe respiratory disease (125). However, it remains uncertain whether APL in COVID-19 patients are transient, as observed in other viral infections, or persistent, which would suggest a long-term thrombotic risk (10). It is hypothesized that SARS-CoV-2 may act synergistically with APL to promote the immunothrombotic process, as APL can directly induce NETosis. Evidence suggests that dysregulated cytokine release in COVID-19 may be sustained by crosstalk between neutrophils and macrophages mediated by NETs, leading to a profoundly exaggerated immunothrombotic response (2).

## 3 Discussion

Immunothrombosis involves a finely tuned interaction between the coagulation system and innate immunity, aimed at preserving host integrity. In COVID-19, this balance becomes disrupted, leading to dysregulated activation of immune and endothelial cells. Dysfunctional endothelial cells adopt procoagulant properties, which, when combined with the hypercoagulable state characteristic of COVID-19, promote the formation of blood clots, further intensifying the immunothrombotic state. This process may involve the activation and elevation of coagulation factors, a diminished function of natural anticoagulants, and impaired fibrinolysis, driven by the upregulation of antifibrinolytic proteins, which further sustain and amplify the immunothrombotic condition.

This interplay may initiate vicious cycles where thrombosis and inflammation reinforce one other. But which comes first – thrombosis or inflammation? While inflammation is widely recognized as a key driver of coagulation activation and increased thrombotic risk, recent findings highlight the reverse relationship, where fibrin, a key component of blood clots, can actively recruit and stimulate inflammatory cells, thereby amplifying inflammation. In COVID-19, inflammation is widely considered the initial trigger, driven by the SARS-CoV-2 virus, where the resulting thrombotic state develops as a secondary consequence.

However, the reverse scenario – thromboinflammation – may become equally plausible, where thrombosis itself exacerbates and sustains the inflammatory response. The therapeutic effects of immune blockers in COVID-19, such as IL-6 inhibitors, have raised questions about whether their benefits stem primarily from suppressing inflammation, reducing hypercoagulation, or a combination of both. Given the bidirectional relationship, as discussed above, it is believable that these agents exert their effects through both mechanisms – dampening the inflammatory response while simultaneously moderating the hypercoagulable state.

Thrombotic complications in COVID-19 demonstrate organ-specific patterns, with distinct mechanisms underlying micro- and macrovascular thrombosis. Pulmonary thrombosis appears to be predominantly immunothrombotic, initiated by local immune responses and direct infection of the endothelium by SARS-CoV-2, which triggers endothelial activation, inflammation, and NET formation, promoting *in situ* thrombosis independent of embolic events. In contrast, systemic thrombosis affecting organs such as the brain, heart, and kidneys, is largely coagulation-driven, characterized by widespread activation of the coagulation cascade, platelet aggregation, and fibrin deposition.

In the management of thromboembolic complications associated with hyperinflammatory diseases, anticoagulant therapy alone has proven insufficient, and combining immunomodulatory and anticoagulant treatments has been shown to significantly improve patient outcomes. In APS, where the immune system plays a central role in the pathophysiology of the thrombotic tendency, the use of immune blockers is often essential to effectively prevent thrombotic events. Immunothrombosis is thought to contribute to resistance to anticoagulant therapy in COVID-19, necessitating the inclusion of immunomodulatory drugs for effective treatment. However, COVID-19-associated coagulopathy is not solely a byproduct of inflammation. Instead, resistance to anticoagulants is likely linked to the thromboinflammatory mechanisms unique to COVID-19. In this context, pre-existing clots act as drivers of thromboinflammation, while the intense inflammatory response diminishes the efficacy of anticoagulant therapies. Thromboinflammation represents a key pathophysiological component of COVID-19, potentially explaining the improved outcomes observed when combining immunomodulatory and anticoagulant therapies. Unlike immunothrombosis, where inflammation activates coagulation without pre-formed clots, thromboinflammation involves pre-existing clots that perpetuate a dysregulated coagulation system in concert with an overactive immune response. This makes thromboinflammation potentially more resistant to treatment and underscores the need for a multifaceted therapeutic approach. Similarly, APS, which can serve as a model for thromboinflammation, shares clinical features of anticoagulation resistance with COVID-19. Both conditions involve complex interactions between innate immune cells and thrombosis, characterized by a systemic proinflammatory and prothrombotic state that cannot be adequately managed with anticoagulants alone. Instead, the inclusion of immunomodulatory therapies is essential for effective treatment. While the evidence is not yet definitive, combining anticoagulants with other agents that target key components of immunothrombosis, such as anti-NET therapies and complement inhibitors, may offer a more effective approach. This multifaceted strategy could help address the complex interplay of coagulation, inflammation, and immune activation, particularly in patients with severe or treatment-resistant disease.

How does ROTEM reflect immunothrombosis, and is it useful in this context? ROTEM provides valuable insights into the hypercoagulable states often associated with immunothrombosis. In this setting, as a reliable marker of hypercoagulation, ROTEM findings typically include shortened clot formation time (CFT) and elevated maximum clot firmness (MCF), indicating rapid clot propagation and increased clot stability. These results align with the hypercoagulable characteristics of the immunothrombotic state.

However, some studies have reported prolonged clotting times (CT) in ROTEM analyses of immunothrombosis, which presents a paradox in the hypercoagulation framework. While prolonged INTEM-CT is often attributed to heparin treatment, prolonged EXTEM-CT cannot be explained by anticoagulation therapy. Instead, it may reflect alterations in extrinsic coagulation pathway factors, such as F VII and tissue factor (TF), which are altered by viral effects and inflammatory activation.

Despite its utility in detecting hypercoagulability, ROTEM has notable limitations in assessing primary hemostasis dysfunctions central to immunothrombosis. It is less effective in evaluating coagulopathy associated with endothelial dysfunction, platelet adhesion, and von Willebrand factor activity – important components of the immunothrombotic process. These limitations are particularly relevant in COVID-19, a condition some researchers characterize as fundamentally an endothelial disorder. In COVID-19, viral invasion and immune cell infiltration drive endothelial dysfunction, platelet activation, and other prothrombotic mechanisms, highlighting the gaps in ROTEM’s ability to fully capture these pathophysiological processes. Therefore, when interpreting ROTEM results in the context of immunothrombosis, it is important to consider these constraints and complement ROTEM with other diagnostic tools for a more comprehensive assessment.

Can knowledge of immunothrombosis and thromboinflammation help us in future pandemics? The answer is very likely yes. A deeper understanding of the complex connections between the coagulation system and the immune system offers valuable insights that extend far beyond the current pandemic. Just as SARS-CoV-2 has demonstrated, future pandemics caused by novel pathogens are likely to involve immune dysregulation and systemic inflammation, leading to thromboinflammatory complications.

With this knowledge, we can enhance our ability to predict, understand, and manage coagulopathy associated with infectious diseases more effectively. This includes identifying at-risk patients, and encourages the development of integrated treatment strategies that address inflammation and thrombosis simultaneously, rather than treating these processes in isolation. Ultimately, the study of immunothrombosis not only prepares us for future pandemics, but also broadens our understanding of the body’s response to infection, opening doors to innovative therapies and preventive measures. This knowledge could shape more resistant healthcare strategies and improve our ability to respond rapidly and effectively to emerging global health threats.

## 4 Conclusion

The relationship between the inflammatory response and the coagulation system is complex, particularly in the context of COVID-19. While ROTEM provides valuable insights into the hypercoagulable states commonly observed in immunothrombosis, it has notable limitations in assessing primary hemostasis dysfunctions. Therefore, a comprehensive assessment of coagulation in immunothrombotic states requires supplementing ROTEM analysis with additional diagnostic tools.

The observed resistance to anticoagulant therapy in COVID-19 likely arises from the state of immunothrombosis and thromboinflammation, where inflammation and coagulation trigger one another in a cyclical manner. This underscores the importance of combining immune-targeted therapies with anticoagulants to optimize treatment outcomes. Notably, parallels can be drawn with antiphospholipid syndrome – another condition marked by immune system activation – where severe thromboembolic disease often necessitates both anticoagulant and immune-modulating therapies for effective management. These comparisons highlight the need for an integrated approach to addressing the dual facets of inflammation and coagulation in hyperimmune disorders.
